# Contrasting Response of Santina and Bing Sweet Cherry Cultivars Under Combined Biotic and Abiotic Stress

**DOI:** 10.3390/plants15030450

**Published:** 2026-02-01

**Authors:** Claudia Carreras, Alan Zamorano, Camila Gamboa, Luis Villalobos-González, Paula Pimentel, Lorena Pizarro, Weier Cui, Manuel Pinto, Carlos Rubilar-Hernández, Analía Llanes, Assunta Bertaccini, Nicola Fiore

**Affiliations:** 1 Departamento de Sanidad Vegetal, Facultad de Ciencias Agronómicas, Universidad de Chile, Santiago 8820808, Chile; claudia.carreras@gmail.com (C.C.); agezac@uchile.cl (A.Z.); camila.gamboa@uchile.cl (C.G.); cuiweierpku@gmail.com (W.C.); 2 Fundación Instituto Profesional Duoc UC, Santiago 7500662, Chile; 3 Programa de Doctorado en Ciencias Silvoagropecuaria y Veterinarias, Campus Sur, Universidad de Chile, Santiago 8820808, Chile; 4 Centro de Estudios Avanzados en Fruticultura (CEAF), Rengo 2940000, Chile; luisvillalobosg1@gmail.com (L.V.-G.); ppimentel@ceaf.cl (P.P.); 5 Instituto de Ciencias Agroalimentarias, Animales y Ambientales, Universidad de O’Higgins, San Fernando 3070000, Chile; lorena.pizarro@uoh.cl (L.P.); manuel.pinto@uoh.cl (M.P.);; 6 Centro de Biología de Sistemas para el Estudio de Comunidades Extremófilas de Relaves Mineros (SYSTEMIX), Universidad de O’Higgins, Rancagua 2820000, Chile; 7 Laboratorio de Fisiología Vegetal-Interacción Planta-Ambiente, Departamento de Ciencias Naturales, Universidad Nacional de Río Cuarto, Ruta Nac. 36—Km. 601, Rio Cuarto, Córdoba X5804BYA, Argentina; allanes@exa.unrc.edu.ar; 8 *Alma Mater Studiorum*—University of Bologna, 40127 Bologna, Italy

**Keywords:** *Pseudomonas syringae* pv. *syringae*, drought stress, *Prunus avium*, transcriptome

## Abstract

Climate change is intensifying the simultaneous occurrence of biotic and abiotic stresses in fruit crops, but yet the molecular mechanisms underlying plant responses remain poorly understood. The physiological and transcriptomic responses of two sweet cherry (*Prunus avium* L.) cultivars, Santina and Bing, grafted onto Gisela 12, were investigated under single and combined stresses imposed by *Pseudomonas syringae* pv. *syringae* and water deficit. Although biomass, gas exchange, and hormone accumulation showed only minor changes, combined stress triggered distinct cultivar-dependent transcriptional reprogramming. The cultivar Bing exhibited a pronounced response with 4261 differentially expressed genes (DEGs), characterized by strong repression of photosynthetic processes and activation of defense- and hormone-related pathways. In contrast, the cultivar Santina showed a moderate response with 674 DEGs, primarily reinforcing structural and secondary metabolism. Cultivar-specific modulation of abscisic acid sensitivity was associated with the contrasting regulation of WRKY40 and Sin3-like repressors, despite comparable ABA levels. Strikingly, both cultivars upregulated the *GIGANTEA* gene, underscoring its role as a central regulatory hub linking circadian rhythm, stomatal function, and hormonal crosstalk under dual stress. Collectively, these results reveal non-additive, genotype-specific transcriptional strategies in sweet cherry trees, providing insights into stress integration in fruit trees and identifying regulatory genes that may inform breeding and management strategies for resilience under climate change.

## 1. Introduction

Plant diseases result when four factors co-occur, known as the disease pyramid: pathogen presence, favorable climatic conditions, a susceptible host, and the time during which these factors converge for the disease to develop. Climate change has led to significant modifications in environmental conditions, one of the four key factors contributing to this disease pyramid, with effects including rising temperatures and alterations in precipitation patterns. Fruit crops are affected by these changes in environmental conditions and, simultaneously, can be stressed by pathogens. To cope with these stresses, plants employ a set of defense strategies, including pathogen-associated molecular pattern-triggered immunity (PTI) and effector-triggered immunity (ETI), which activate local and systemic defenses to limit pathogen colonization [1].

Sweet cherry (*Prunus avium* L.) is the most extensively planted fruit tree species in Chile, covering 63,494.87 ha [2], and is of high economic importance worldwide [3]. Its establishment and productivity are challenged by both abiotic and biotic stresses, notably water deficit and bacterial canker caused by *Pseudomonas syringae* pv. *syringae*. The production losses associated with *P. syringae* pv. *syringae* are about 10–40% in commercial orchards [4]. Susceptibility varies markedly among cultivar–rootstock combinations, with Bing being among the most susceptible cultivars [5], whereas Santina generally exhibits lower susceptibility [5,6,7]. Gisela rootstocks display variable responses to drought and pathogen stress depending on the scion combination, with Gisela 12 more adapted for regulated deficit irrigation than other Gisela rootstocks under water deficit [8]. *P. syringae* is a hemibiotrophic pathogenic bacterium with a vast host range, relying on a complex arsenal of type III effectors, phytotoxins, and phytohormone manipulation to suppress plant immunity and promote colonization [9]. Among the phytohormones involved in plant defense, salicylic acid (SA) plays a pivotal role in resistance against hemibiotrophs and biotrophs pathogens and is also implicated in tolerance to abiotic stresses, including salinity and drought [10,11,12,13]. Conversely, drought responses are largely orchestrated by abscisic acid (ABA), which regulates stomatal closure, antioxidant defenses, and osmotic adjustment [14,15]. Crosstalk between SA and ABA can be either antagonistic or synergistic, depending on typology of stress, intensity, and timing, thereby shaping plant susceptibility or tolerance under stress conditions [15,16,17,18].

In agricultural environments, biotic and abiotic stresses often occur simultaneously, producing non-additive effects and unique transcriptional and physiological profiles that cannot be predicted from individual stress responses [19,20]. To date, most knowledge on plant responses to multifactorial and combined stresses, such as drought interacting with heat or pathogen infection, has been generated from studies in *Arabidopsis thaliana*; (L.), Heynh. (plant used as a model) and annual crops, including rice and maize, whereas comparable integrative analyses remain scarce in perennial fruit trees [20,21,22,23,24]. Unlike annual plants, fruit trees must balance long-term carbon allocation among structural growth, fruit production, and defense mechanisms across multiple growing seasons, which may fundamentally shape their stress response strategies. Consequently, stress responses in woody perennials are influenced not only by rapid defense activation but also by sustained metabolic and regulatory adjustments that can differ from those described in herbaceous species [7,25].

The outcome strongly depends on stress severity, sequence, and duration [20]. For example, pathogen infection can reduce photosynthesis and water-use efficiency (WUE) and alter stomatal dynamics, thereby influencing plant tolerance to subsequent drought [26]. The timing of drought relative to pathogen infection can determine the magnitude and even the direction of the interaction, with early-season biotic stress sometimes exacerbating later drought effects, and vice versa [20,24,26].

In Chilean fields, sweet cherry trees often experience *P. syringae* pv. *syringae* infection during late winter and early spring, followed by water deficit in summer due to prolonged drought and postharvest irrigation practices [5,9,27,28]. Under these conditions, cultivar differences in susceptibility to *P. syringae* pv. *syringae* and drought resilience may lead to contrasting adaptive strategies under combined stress [6,29]. Studies in model plants and annual crops have shown that combined stress triggers genotype-specific responses, involving both shared and unique sets of differentially expressed genes [19,20], but such integrative physiological–transcriptomic analyses remain scarce in perennial fruit trees. Recent studies have underscored the role of integrative regulatory hubs, such as GIGANTEA (GI), which function at the interface of circadian rhythm, chloroplast development, metabolite accumulation, and ABA signaling, thereby influencing both drought tolerance and pathogen defense [30,31]. These multifunctional regulators exemplify how plants coordinate complex physiological and molecular processes to adapt to variable and often concurrent environmental challenges. However, the extent to which such regulatory pathways are modulated under combined biotic and abiotic stresses remains poorly understood in perennial fruit crops, particularly in sweet cherry cultivars exhibiting contrasting susceptibility to bacterial canker [19,20]. Elucidating these mechanisms, including the potential crossregulation of hormonal and metabolic networks, is critical for developing targeted breeding, agronomic management strategies, or biotechnological approaches that ensure sustainable fruit production under changing climatic conditions.

At the cellular level, both biotic stress and water deficit ultimately impose constraints on primary metabolism, requiring plants to rapidly reprogram energy allocation and resource use. Increasing evidence indicates that such metabolic reprogramming in plants involves a stringent-like response, in which chloroplasts act as central regulatory hubs [32,33]. This response is mediated by the accumulation of atypical alarmone nucleotides [(p)ppGpp], which modulate transcriptional and translational activity and downregulate photosynthetic and growth-related processes under stress conditions [34,35,36,37].

Based on these considerations, it was hypothesized that combined *P. syringae* pv. *syringae* infection and water deficit would trigger distinct and non-additive transcriptional responses in sweet cherry cultivars with contrasting susceptibility to bacterial canker, associated with differential regulation of stress and defense-related pathways. The present study aimed to evaluate the physiological and transcriptomic responses of two sweet cherry cultivars, Santina and Bing, grafted onto Gisela 12, when subjected to single (*P. syringae* pv. *syringae* or water deficit) and combined stresses. The objectives were to identify differentially expressed genes and enriched pathways associated with biotic, abiotic, and combined stress responses; and to uncover cultivar-dependent regulatory mechanisms, including potential transcriptional hubs, which could explain their contrasting susceptibility to bacterial canker and drought. By integrating physiological and molecular analyses, this study provides a framework for understanding stress interaction in sweet cherry.

## 2. Results

### 2.1. Infection Monitoring

At 49 days post-inoculation (dpi), the presence of *P. syringae* pv. *syringae* (Pss11116B1) was confirmed in all inoculated treatments (three plants per treatment) based on the growth of fluorescent colonies on PAF (*Pseudomonas* agar F) culture medium. This confirmation was obtained before the implementation of differential irrigation regimes. No fluorescent colonies were detected in the mock treatments (T2 and T4) or in samples from zone P1 (leaf petiole above the inoculation point) in any treatment. In contrast, zones P2 and P3 (above and below the inoculation site) showed consistent colonization, with cultivar Bing exhibiting significantly higher colony counts than cultivar Santina (n = 9; *p* < 0.0001). PCR amplifications of the *syrB* and *syrD* genes from fluorescent colonies confirmed their identity as *P. syringae* pv. *syringae*, supporting the success of the inoculation method and the local progression of the infection within the stem tissues.

Despite the successful detection of *P. syringae* pv. *syringae* at 49 dpi, no bacterial transcripts were detected in any treatment at 124 dpi when mapping the *de novo* assembled RNA-seq reads to the Pss11116B1 reference genome (GenBank accession number GCA_029383325.1). This likely reflects reduced bacterial activity or population density at this stage.

### 2.2. Physiological Response

No significant differences were observed between *P. syringae* pv. *syringae* and mock treatments for stomatal conductance (g_s_; *p* = 0.764), net assimilation (A; *p* = 0.376), transpiration (E; *p* = 0.647), or water-use efficiency (WUE; H = 1.00, *p* = 0.267).

**Figure 1:**
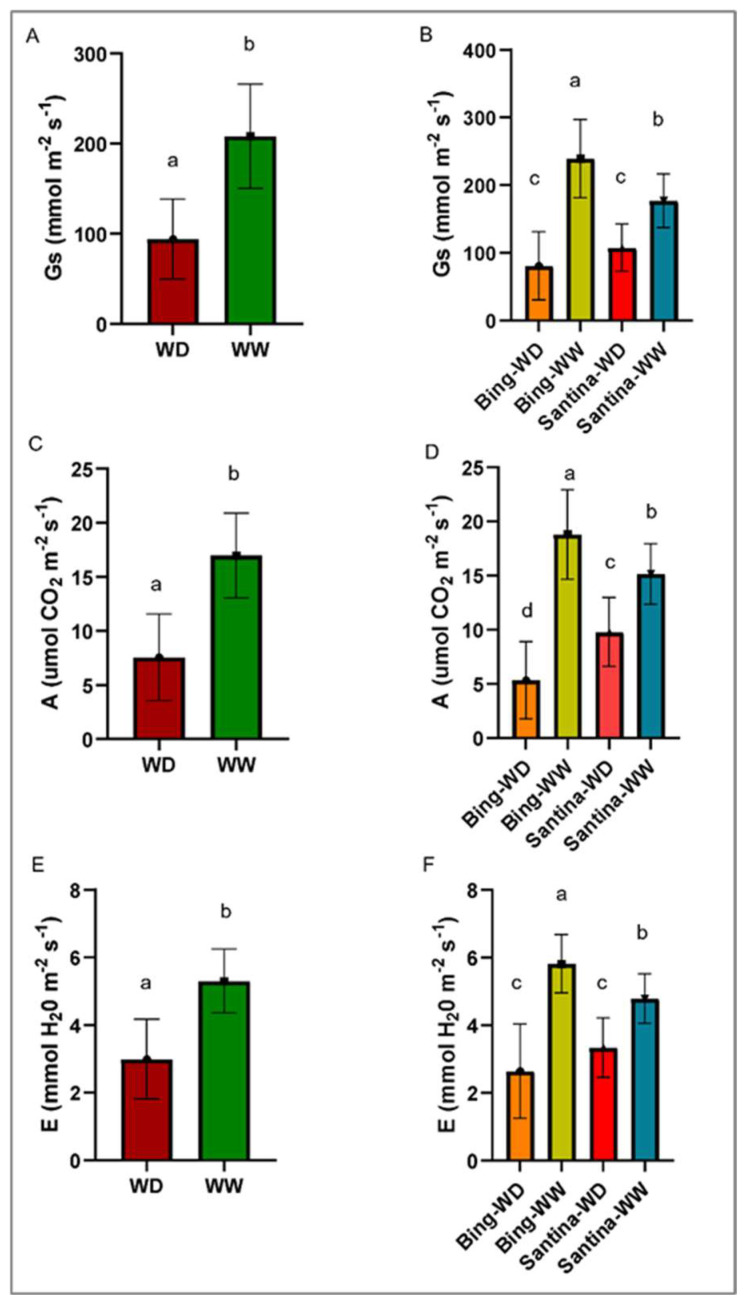
Analysis of physiological variables in different cherry treatments at 124 dpi, considering Santina-Bing cultivars, inoculation treatments, and irrigation regimes (WW—well-watered; WD—water deficit). (**A**,**B**) Stomatal conductance (Gs; mmol m^−2^ s^−1^); (**C**,**D**) net assimilation (A; μmol CO_2_ m^−2^ s^−1^); (**E**,**F**) transpiration (E; mmol H_2_O m^−2^ s^−1^). Bars indicate means, and vertical lines represent ± SD. Different letters indicate statistically significant differences according to Fisher’s LSD test (*p* < 0.05). Note: Each column in (**A**,**C**,**E**) includes both varieties.

Under water deficit (WD), the trees reached a moderate level of water deficit, characterized by leaf water potential (Ψ) of –1.3 MPa and stomatal conductance (g_s_) < 100 mmol m^−2^ s^−1^. Bing trees under well-watered (WW) conditions (Bing-WW) exhibited the highest g_s_, assimilation (A), and transpiration (E) (*p* = 0.019; *p* = 0.017 and *p* = 0.0026) (Figure 1). Both cultivars showed reduced photosynthetic performance under WD.

Finally, the interaction between biotic and abiotic stress was evaluated. No significant interactions between *P. syringae* pv. *syringae* and WD were detected for gₛ (*p* = 0.626), A (*p* = 0.666), or E (*p* = 0.646).

### 2.3. Biomass Accumulation

**Figure 2:**
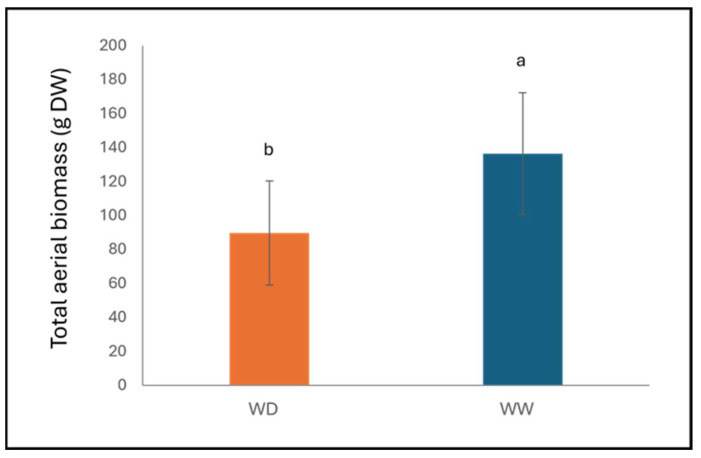
Biomass at 124 dpi in cvs. Santina and Bing plants under different irrigation regimes (WW—well-watered; WD—water deficit). The bars indicate the standard deviation. Bars indicate means, and vertical lines represent ± SD (n = 6). Different letters indicate statistically significant differences according to Fisher’s LSD test (*p* < 0.05).

No significant differences in aerial, root, leaf, or stem biomass were detected between plants inoculated with *P. syringae* pv. *syringae* and mock controls. In contrast, irrigation had a distinct effect on plant growth; total aerial biomass (n = 3 per treatment) was significantly higher in WW trees compared with WD ones (*p* = 0.009; Figure 2). Under combined stress, biomass was not significantly affected (total aerial: *p* = 0.141; root: *p* = 0.285; leaf: *p* = 0.372; stem: *p* = 0.354).

### 2.4. Hormonal Response

**Figure 3:**
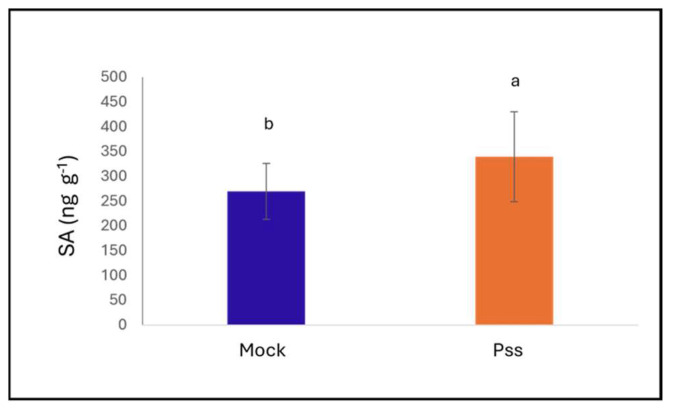
SA levels in leaves of sweet cherry trees. SA levels at 1 dpi between mock and *P. syringae* pv. *syringae* treatments. SA levels in grams of dry weight. Bars indicate means, and vertical lines represent ± SD (n = 6). Different letters indicate statistically significant differences according to Fisher’s LSD test (*p* < 0.05).

SA levels increased significantly after *P. syringae* pv. *syringae* inoculation (*p* = 0.036). At 1 dpi, cv. Santina showed higher accumulation of JA and ABA than cv. Bing, although these differences were not statistically significant. At 124 dpi, SA tended to remain higher in inoculated plants (+30 ng g^−1^ DW), but the difference was still not significant (*p* = 0.0734) (Figure 3).

**Figure 4:**
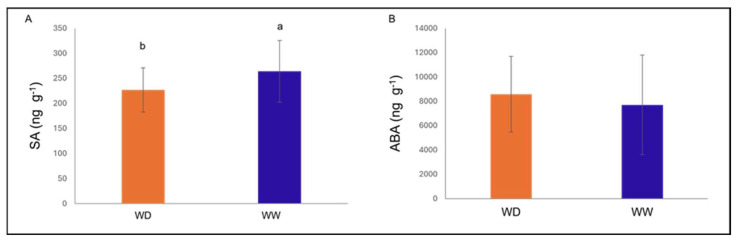
Hormone content in cvs. Santina and Bing plants under different irrigation regimes at 124 dpi. (**A**) Salicylic acid (SA, ng g^−1^ dry weight [DW]) and (**B**) abscisic acid content (ABA, ng g^−1^ dry weight [DW]). WD—water deficit; WW—well-watered. Bars indicate means, and vertical lines represent ± SD (n = 6). Different letters indicate statistically significant differences according to Fisher’s LSD test (*p* < 0.05).

At 124 dpi, SA content was significantly higher in WW trees compared with WD plants (+37 ng g^−1^ DW; *p* = 0.032). No significant effects of irrigation were observed for other hormones measured; ABA tended to be higher under WD, but differences were not significant (Figure 4). Although ABA concentrations did not differ significantly among treatments at 124 dpi, transcriptomic analyses revealed differential expressions of ABA metabolic genes under combined stress conditions (Section 2.5.3). Under combined stress, hormone levels (ABA, JA, SA, IAA, and GA_3_) were not significantly affected (*p* > 0.05).

### 2.5. Transcriptomic Analysis

**Figure 5:**
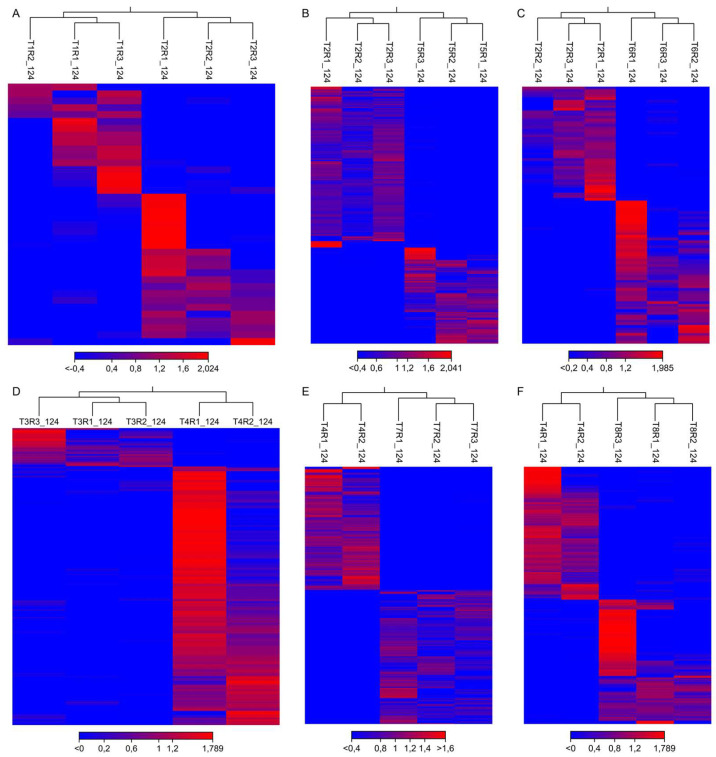
Heatmap of differentially expressed genes at 124 dpi. Each panel represents a pairwise comparison between stressed and mock conditions: (**A**) cv. Santina—Pss (T1 vs. T2); (**B**) cv. Santina—combined stress (T5 vs. T2); (**C**) cv. Santina—water deficit (T6 vs. T2); (**D**) cv. Bing—Pss (T3 vs. T4); (**E**) cv. Bing—combined stress (T7 vs. T4); and (**F**) cv. Bing—water deficit (T8 vs. T4). Gene expression is displayed as fold change, with clustering based on Euclidean distance. R1–R3 represent biological replicates. Treatments T1–T8 are described in detail in the experimental design.

At 124 dpi, raw libraries yielded between 50.2 and 71.9 million paired-end reads per sample (n = 24; mean ± SD: 59.9 ± 6.5 M reads), with GC content ranging from 44.9% to 49.1% (mean: 46.4%) (Table S1). Base-calling accuracy was high across all libraries, with Q20 scores ≥ 96.0% and Q30 scores ≥ 90.5%. After adapter and quality trimming (error probability threshold 0.01; maximum two ambiguous bases; minimum length 50 nt), an average of 97.9% of reads per library were retained. Post-trimming sequencing depth ranged from 49.1 to 70.5 million reads per sample, ensuring robust coverage for differential expression analysis. Trimmed reads were aligned to the *Prunus avium* cv. Satonishiki reference transcriptome (GenBank accession number GCF_002207925.1_PAV_r1.0), with an average mapping rate of 37.25%. Hierarchical clustering at 124 dpi confirmed consistent grouping of biological replicates, except for replicate T4R3 (Bing, mock under well-watered conditions), which did not cluster with its treatment group in hierarchical clustering, was therefore considered an outlier, and thus excluded from all subsequent analyses (Figure 5).

**Figure 6:**
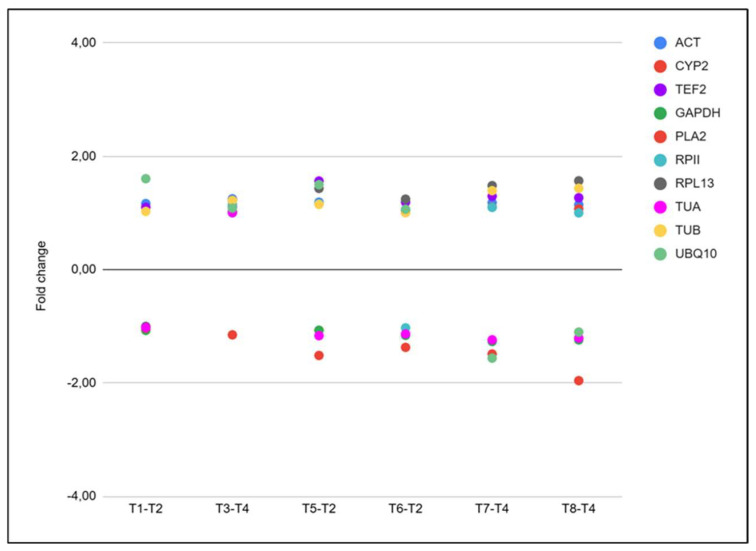
Expression levels (fold change) of housekeeping genes at 124 dpi across different treatment comparisons. Each dot represents the fold change value of one housekeeping gene between pairs of treatments: T1–T2, T3–T4, T5–T2, T6–T2, T7–T4, and T8–T4. Gene expression was evaluated for *ACT*, *CYP2*, *TEF2*, *GAPDH*, *PLA2*, *RPII*, *RPL13*, *TUA*, *TUB*, and *UBQ10*. Color coding corresponds to each gene, as indicated in the legend. Treatments T1–T8 are described in detail in the experimental design.

The cutoff for differentially expressed genes (DEGs) was FC| ≥ 2, and FDR < 0.05. Expression of reference housekeeping genes [38] remained stable (|FC| < 2) across all treatments, confirming the reliability of the RNA-seq data (Figure 6).

To validate RNA-seq expression profiles, fold change values obtained by RNA sequencing were compared with RT-qPCR results for selected differentially expressed genes. Spearman’s rank correlation analysis revealed a significant positive correlation between RNA-seq and qPCR fold change values (ρ = 0.64, *p* = 0.013).

#### 2.5.1. Responses to Biotic Stress (*P. syringae* pv. *syringae*)

**Figure 7:**
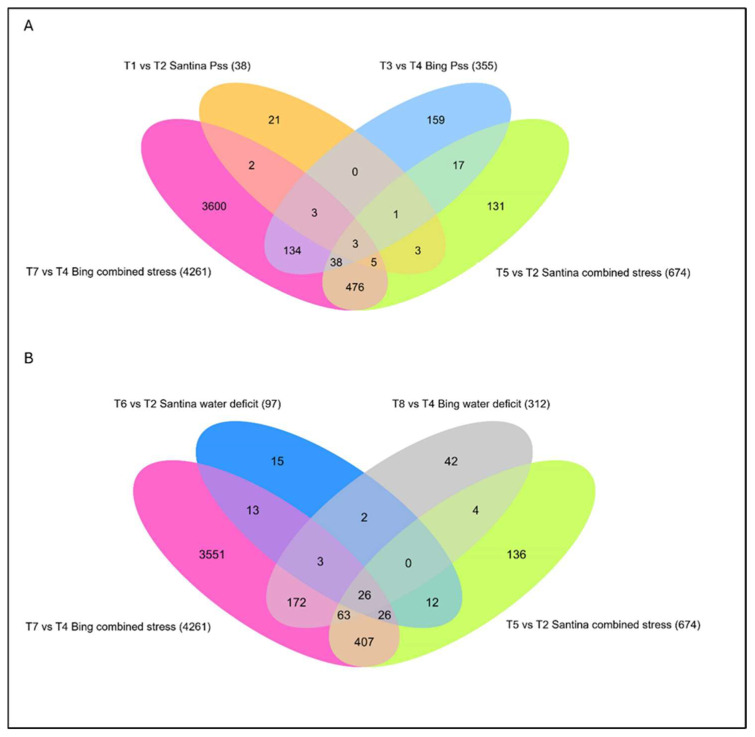
Venn diagrams show the number of unique and shared differentially expressed genes (DEGs) in sweet cherry cultivars Santina and Bing under biotic, abiotic, and combined stress conditions at 124 dpi. (**A**) DEGs under biotic stress (T1 vs. T2 and T3 vs. T4) and combined stress (T5 vs. T2 and T7 vs. T4). (**B**) DEGs under water deficit (T6 vs. T2 and T8 vs. T4) and combined stress (T5 vs. T2 and T7 vs. T4). Each ellipse represents a specific pairwise comparison, and the overlaps indicate the number of shared DEGs among conditions. Numbers within each sector indicate the exact number of DEGs detected in each pairwise comparison. Treatments T1–T8 are described in detail in the experimental design.

In both cultivars, combined stress (T5 and T7) induced more DEGs than single stresses, either biotic (T1, T3) or abiotic (T6, T8), relative to mock controls (T2 and T4) (Figure 7).

**Figure 8:**
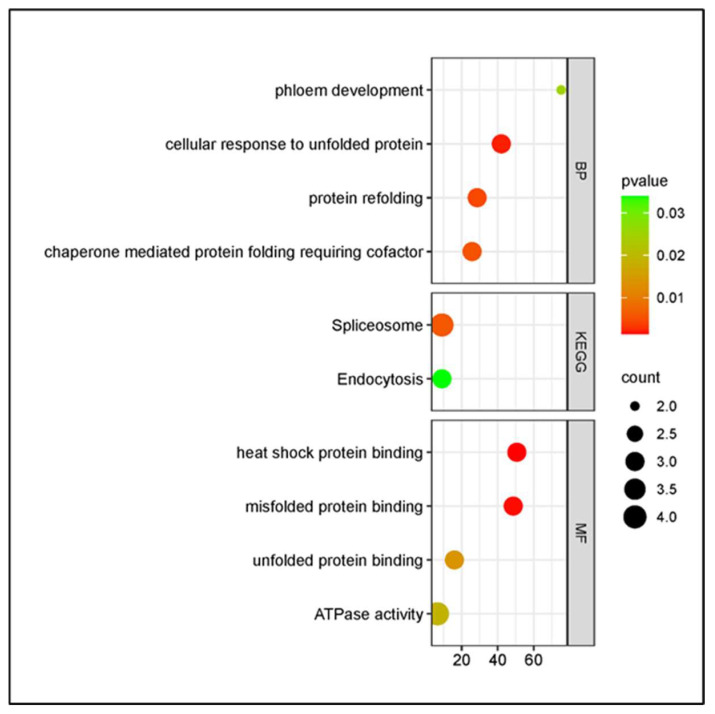
Gene Ontology (GO) and KEGG pathway enrichment analysis of differentially expressed genes (DEGs) at 124 dpi in sweet cherry cv. Santina in response to *P. syringae* pv. *syringae* (Pss11116B1) inoculation. Enriched terms are grouped by category: BP (Biological Processes), MF (Molecular Functions), and KEGG (Kyoto Encyclopedia of Genes and Genomes pathways). Dot size indicates the number of DEGs associated with each term, and color represents statistical significance (*p*-value). Only significantly enriched terms (*p* < 0.05) are shown.

Transcriptomically, cv. Santina-*P*. *syringae* pv. *syringae* (T1) displayed fewer DEGs than cv. Bing-*P. syringae* pv. *syringae* (T3) (Figure 8).

**Figure 9:**
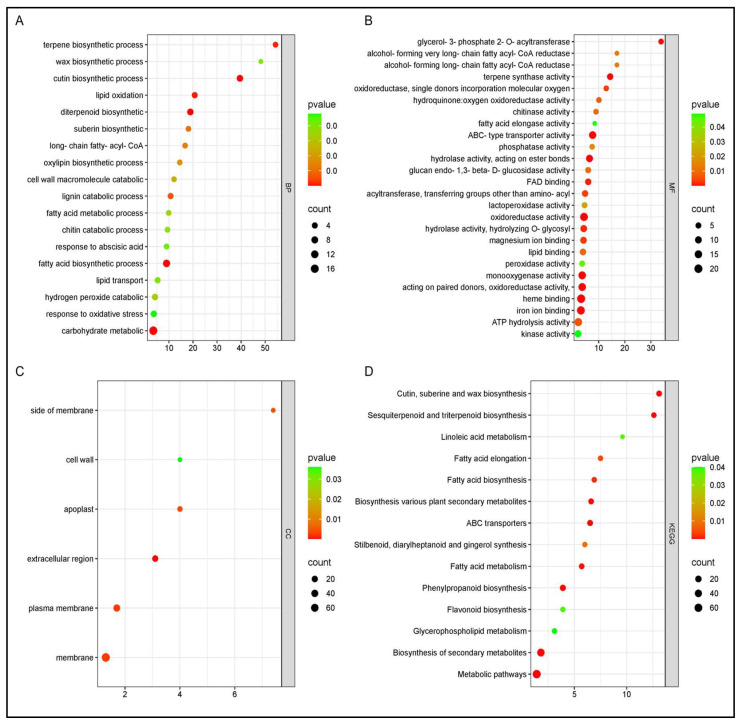
Gene Ontology (GO) and KEGG pathway enrichment analysis of differentially expressed genes (DEGs) at 124 dpi in sweet cherry cv. Bing in response to *P. syringae* pv. *syringae* (Pss11116B1) inoculation. (**A**) Biological Processes (BP), (**B**) molecular Functions (MF), (**C**) cellular Components (CC), and (**D**) Kyoto Encyclopedia of Genes and Genomes (KEGG) pathways. Dot size represents the number of DEGs associated with each term, while dot color indicates the significance level based on the adjusted *p*-value. Only terms with *p* < 0.05 are displayed.

Functional enrichment analyses revealed contrasting late-stage strategies between cultivars under *P. syringae* pv. *syringae* infection, involving stress-related protein homeostasis in cv. Santina and repression of structural barrier biosynthesis in cv. Bing (Figure 9).

Analysis of SA-pathway genes [39,40,41] showed no DEGs in cv. Santina, while cv. Bing displayed downregulation of PR4-related proteins and phenylalanine ammonia-lyase (PAL) (Table S2). Comparison of *P. syringae* pv. *syringae*-inoculated and combined-stress treatments identified three shared DEGs: those involved in diterpenoid biosynthesis, which were upregulated in all treatments, and those linked to carbohydrate metabolic processes, which were downregulated across all treatments.

#### 2.5.2. Response to Abiotic Stress (WD)

**Figure 10:**
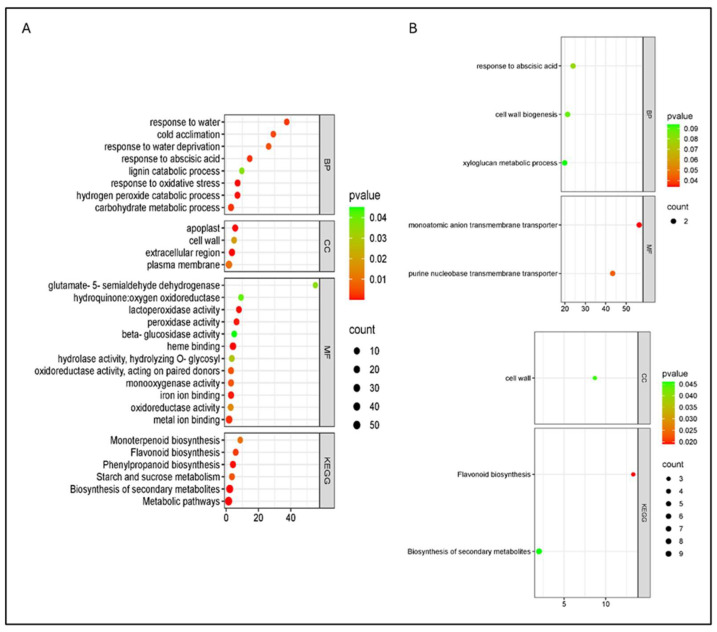
Gene Ontology (GO) and KEGG pathway enrichment analysis of differentially expressed genes (DEGs) in sweet cherry under water deficit (WD) at 124 dpi. (**A**) Bing; (**B**) Santina. BP—biological processes, CC—cellular components, MF—molecular functions, and KEGG—Kyoto Encyclopedia of Genes and Genomes pathways. Dot size represents the number of DEGs associated with each term, while dot color indicates the significance level based on the adjusted *p*-value. Only terms with *p* < 0.05 are displayed.

Under water deficit, both cultivars showed transcriptional changes dominated by ABA-related signaling and metabolic adjustment, with a stronger and more diverse response in cv. Bing (312 vs. 97). Bing showed enrichment in eight additional biological processes, most notably “response to water deprivation”. Consistent with GO enrichment, pathway-level analyses highlighted the modulation of secondary metabolism, including monoterpenoid and flavonoid biosynthesis, with most associated genes downregulated. In cv. Santina, enriched processes included the cell-wall polysaccharide biosynthetic process and xyloglucan metabolic process, with all related genes downregulated (Figure 10). A (+)-neomenthol dehydrogenase gene was upregulated in cv. Santina and downregulated in cv. Bing. WD and combined stress shared 26 DEGs (glycine-rich proteins, LEA proteins, and cold-induced proteins), which were upregulated in all conditions.

#### 2.5.3. Response of Combined *P. syringae* pv. *syringae* and Water Deficit

To assess whether combined biotic and abiotic stress elicits emergent transcriptional responses beyond those induced by individual stresses, it was compared the combined-stress response to single-stress conditions. The cv. Bing under combined stress (T7) exhibited a dramatic transcriptomic response with 4261 DEGs; cv. Santina (T5) had only 674 DEGs (Figure 7). Analysis of DEG overlap between combined stress and single stress revealed low convergence, indicating cultivar-specific transcriptional programs. In cv. Bing, only 6.2% of combined-stress DEGs overlapped with WD (264 DEGs), and the overlap with *P. syringae* pv. *syringae* alone was similarly low (4.2%); in cv. Santina, the overlap was also limited compared with *P. syringae* pv. *syringae*, but there were more DEGs between combined stress and WD (13.79%).

**Figure 11:**
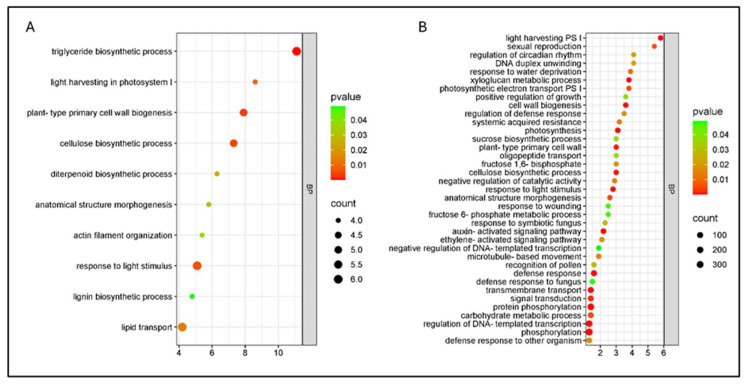
Gene Ontology (GO) enrichment analysis of differentially expressed genes (DEGs) in sweet cherry cvs. Santina and Bing under combined stress (Pss11116B1 and WD) at 124 dpi. (**A**) Santina (T5); (**B**) Bing (T7). Enrichment results correspond to the Biological Processes (BP) category. Dot size represents the number of DEGs associated with each term, while dot color indicates the significance level based on the adjusted *p*-value. Only terms with *p* < 0.05 are displayed.

Enrichment analyses revealed sharply divergent functional strategies between cultivars under combined stress (Figure 11).

**Figure 12:**
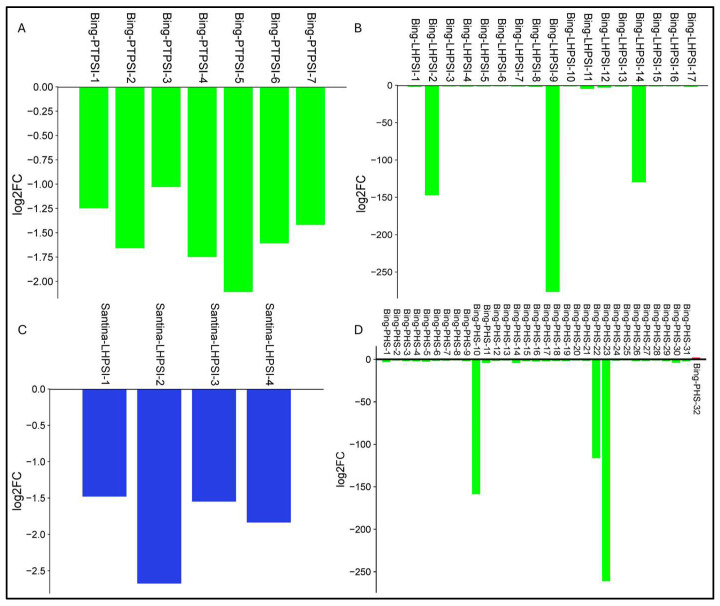
Fold change in genes associated with photosynthesis-related processes under combined stress at 124 dpi in sweet cherry cultivars. (**A**) Differential expressions of genes involved in photosynthetic electron transport in photosystem I (PTPSI) in cv. Bing. (**B**) Differential expression of genes associated with light harvesting in PSI (LHPSI) in cv. Bing. (**C**) Differential expression of genes associated with light harvesting in PSI (LHPSI) in cv. Santina. (**D**) Differential expression of genes associated with photosynthesis (PHS) in cv. Bing. Bars represent individual differentially expressed genes, plotted as log_2_ FC relative to the corresponding well-watered mock control.

In the cv. Bing, photosynthesis-related processes (light harvesting, light harvesting in PSI, and photosynthetic electron transport in PSI) were downregulated (Figure 12), while defense- and hormone-signaling terms were mostly upregulated. In cv. Santina, repression was restricted to light harvesting in PSI, with other photosynthetic processes not enriched, and structural/secondary metabolism terms showing mixed regulation (Figure 11).

Both cultivars shared 522 DEGs under combined stress, enriched in photosynthesis, cell wall biogenesis, lipid metabolism, and polysaccharide catabolism. The cv. Bing showed stronger negative fold changes in photosynthetic and structural genes, while cv. Santina exhibited milder repression. For example, XP_02184030, XP_02185263, and XP_02185254 were downregulated in cv. Bing (–2.68 to –8.80 FC) but only moderately in cv. Santina (–2.73 to –3.75 FC). Unique DEGs included NPR4-like ankyrin repeat proteins (upregulated in cv. Bing under combined stress but repressed under *P. syringae* pv. *syringae* alone), PR4 (repressed in cv. Bing-*P. syringae* pv. *syringae*, upregulated in cv. Bing combined stress), and a Sin3-like protein (upregulated in cv. Santina, downregulated in cv. Bing). Defense-related PR genes were broadly repressed in cv. Bing (T3, T7), with PR1-like repressed in both cultivars.

Under combined stress conditions in cv. Bing (T7), some genes associated with ABA metabolism were differentially expressed. A zeaxanthin epoxidase gene, involved in the early steps of ABA biosynthesis, was downregulated (FC = −1.61). In parallel, genes encoding ABA catabolic enzymes showed contrasting regulation: abscisic acid 8′-hydroxylase 1-like was strongly upregulated (FC = +5.51), whereas abscisic acid 8′-hydroxylase 4 was downregulated (FC = −2.65). Additionally, an abscisic-aldehyde oxidase-like gene, catalyzing the last step of ABA biosynthesis, displayed a modest upregulation (FC = +1.25). Particularly, no differential expression was detected for 9-cis-epoxycarotenoid dioxygenase (NCED) genes. WRKY22, WRKY40, and WRKY53 showed repression under *P. syringae* pv. *syringae* alone but induction under combined stress in cv. Bing, indicating stress-specific transcriptional shifts. Also, a gene encoding an NB-ARC domain-containing disease resistance protein was downregulated in cv. Santina but upregulated in cv. Bing.

**Figure 13:**
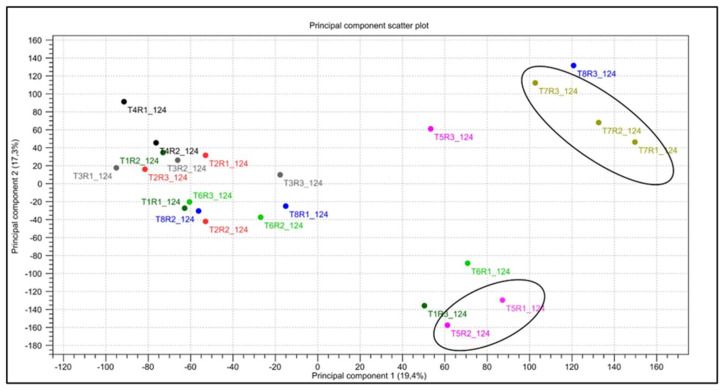
Principal component analysis (PCA) scatter plot (2D) of transcriptome profiles from sweet cherry trees under different stress treatments at 124 dpi. Each point represents an individual biological replicate. Samples are colored by treatment group (T1–T8), and ellipses highlight the clustering of biological replicates under combined stresses. Treatment details are provided in the experimental design section.

Multivariate analysis confirmed that the combined-stress response represents a distinct transcriptional state, particularly in cv. Bing (Figure 13). The first principal component (PC1) accounted for 19.4% of the total variance and was predominantly influenced by the transcriptomic profile of cv. Bing under combined stress (T7), while the second principal component (PC2) explained 17.3% of the total variance. The top 100 genes contributing to this component were significantly enriched for GO terms related to photosynthesis. Furthermore, examination of the top contributors to PC1 revealed that DEGs encoding drought-responsive proteins, transcription factors such as basic leucine zipper (bZIP) (upregulated in T7), Myb-related proteins (downregulated in both T5 and T7), photosynthetic apparatus components, and transport–signal transduction proteins (mitochondrial carnitine/acylcarnitine transporter-like protein) drove the separation along this axis.

## 3. Discussion

This study examined the transcriptional and physiological responses of two sweet cherry cultivars, Santina and Bing, which differ in their susceptibility to bacterial canker (*P. syringae* pv. *syringae*), with cv. Bing being more susceptible [6]. The focus on combined biotic and abiotic stress is especially relevant because plants are regularly subjected to this combination of stresses in the field, and combined responses can be non-additive and cannot be inferred from individual stress responses alone [20,42]. Here, combined drought and *P. syringae* pv. *syringae* elicited cultivar-dependent transcriptional responses, whereas physiological interaction effects were limited at the measured time point, consistent with molecular reprogramming preceding detectable physiological shifts [43,44].

The average RNA-seq mapping rate (~37%) reflects the use of a cultivar-independent reference transcriptome and the high genetic diversity typical of fruit trees. Accordingly, possible interpretations emphasize robust within-cultivar differential responses and comparative, pathway-level patterns between cultivars, rather than absolute transcriptome completeness or gene-specific causal inference.

Regarding the pathogen colonization and persistence, Pss11116B1 was detected at 49 dpi, in all inoculated treatments, indicating localized colonization near the inoculation site. The absence of colonies in zone P1 (above the inoculation point) confirmed spatial restriction, consistent with previous findings in *Prunus* twigs [45]. By 124 dpi, RNA-seq revealed no *P. syringae* pv. *syringae* transcripts in any treatments, which likely reflects reduced bacterial metabolic activity or population density under hight summer temperatures that suppress symptom development [9,27,28,46]. Thus, the absence of detectable bacterial transcripts at 124 dpi does not exclude sustained host defense reprogramming triggered by earlier pathogen perception. The transcriptomic analysis was conducted at this late time point to capture host-driven transcriptional adjustments associated with long-term pathogen colonization and sustained water deficit. However, the use of a single late sampling point limits the resolution of temporal dynamics; future studies including intermediate sampling points will fully characterize response progression.

In response to *P. syringae* pv. *syringae,* cv. Bing exhibited a higher number of DEGs (355) compared to cv. Santina [38]. While resistant varieties often exhibit stronger early transcriptional activation [47,48,49], DEG profiles change over time after inoculation [50,51]. At 124 dpi, inoculation did not result in detectable alteration in growth or gas exchange in either cultivar. Similar findings were reported previously, where cv. Bing showed no changes in transpiration at 90 dpi, whereas cv. Santina exhibited reductions in stomatal conductance and net CO_2_ assimilation under infection [7]. Biomass measurements were limited due to destructive sampling in woody perennials; no biomass reduction was detected, suggesting no major short-term biomass penalty at this stage. Nonetheless, limited power may reduce the sensitivity to subtle effects, type II error cannot be excluded, and cumulative impacts may emerge in subsequent seasons.

Both cultivars accumulated higher salicylic acid (SA) levels in inoculated plants than in controls, aligning with SA-mediated defense against hemibiotrophic pathogens [13,52]. At 1 dpi, cv. Santina tended to show higher JA and ABA levels than cv. Bing, suggesting an early, multi-hormonal response. Although SA and JA are often considered antagonistic, their co-accumulation has been reported in effector-triggered immunity [13,15,16,53,54,55] and may contribute to the reduced susceptibility of cv. Santina. By 124 dpi, ABA concentrations did not differ significantly; however, some ABA-related transcriptional regulators (WRKY22, WRKY40, WRKY53, bZIP TF, and Sin3-like protein) showed a cultivar-dependent expression. In cv. Bing, under combined stress, ABA metabolic genes were differentially expressed, including repression of zeaxanthin epoxidase, modest upregulation of abscisic-aldehyde oxidase, and strong induction of abscisic acid 8′ [56]. Together, these patterns suggest the regulation of ABA turnover and signaling sensitivity rather than sustained ABA biosynthesis [57]. Hormone measurements showed substantial biological variability, and limited replication may have constrained detection of treatment effects.

At the transcriptomic level, cv. Santina upregulated protein refolding and unfolded protein response genes, consistent with maintenance of protein homeostasis [58]. The cv. Bing, in contrast, exhibited a downregulation of genes associated with the biosynthesis of cuticular wax, suberin, and cutin biosynthesis, potentially weakening structural defenses [59]. Because this study did not include anatomical or biochemical measurements of cuticle traits, these inferences remain transcriptome-based. Cuticle-related transcriptional shifts may precede measurable anatomical changes or affect wax composition, which strongly influences non-stomatal water loss [60,61].

Under water deficit, both cultivars grafted on moderately drought-tolerant Gisela 12 [8,62] showed physiological changes consistent with moderate water deficit, including lower leaf water potential and stomatal conductance [63]. CO_2_ assimilation decreased, but cv. Santina outperformed cv. Bing in WD conditions, suggesting greater photosynthetic resilience despite similar biomass outcomes [64]. WD transcriptomes showed the enrichment of ABA-responsive processes and repression of photosynthesis-related genes, while ABA levels tended to increase under WD [65,66], consistent with variable ABA dynamics across tree species [67]. A (+)-neomenthol dehydrogenase gene was upregulated in cv. Santina, but downregulated in cv. Bing, aligning with cultivar-dependent regulation of terpenoid-related stress responses [68,69]. Both cultivars upregulated DREB/CBF (dehydration-responsive element-binding) transcription factors under WD [70,71], whereas AP2/ERF (APETALA2/ethylene-responsive) were downregulated; given the role of some AP2/ERFs in wax biosynthesis [72], this may indicate limited reinforcement of cuticle-related traits during prolonged WD.

When both stresses acted simultaneously, responses were clearly non-additive and cultivar-dependent. Combined stress elicited more DEGs than single stresses in both cultivars [20,23,42], with cv. Bing exhibiting extensive reprogramming, and fewer DEGs overlapped with single-stress responses, indicating an emergent transcriptional profile. Enrichment analyses revealed divergent strategies: in cv. Bing, photosynthesis-related categories were exclusively downregulated, whereas defense and hormone-associated processes were predominantly upregulated, consistent with a shift away from primary metabolism toward defense signaling. In cv. Santina, photosynthetic repression was limited to PSI light harvesting, while lignin and diterpenoid biosynthesis processes showed mixed regulation, consistent with a more targeted adjustment potentially linked to its earlier response to infection [25].

Such transcriptome-level repression of photosynthetic functions under biotic stress, water deficit, and their combination is consistent with active metabolic reprogramming rather than passive damage. In plants, similar patterns have been linked to a chloroplast-centered stringent-like mechanism in which (p)ppGpp alarmones suppress photosynthetic functions and redirect resources toward acclimation [34,36]. Although (p)ppGpp was not measured, the coordinated downregulation of photosynthesis-related genes aligns with chloroplast-centered models described under abiotic and biotic stress conditions. Interpretations in this study are based on differential expression patterns and functional annotations inferred from sequence homology and curated databases. While core stress-response pathways are broadly conserved, species-specific regulatory differences are expected, particularly in woody perennials; therefore, it is possible to emphasize pathway-level patterns rather than definitive causal roles of individual genes. One biological replicate from the cv. Bing well-watered control was excluded based on quality control (hierarchical clustering); despite reduced replication in that group, consistent clustering and differential expression patterns across remaining samples support the robustness of the main conclusions.

At the transcription factor level, WRKY induction under combined stress was observed in both cultivars, with stronger induction in cv. Bing. WRKY40, a negative regulator of ABA signaling in *A. thaliana* [73], was upregulated in cv. Bing, whereas a Sin3-like repressor showed opposite regulation between cultivars. These patterns suggest cultivar-specific repression strategies that may fine-tune ABA sensitivity, although functional roles in sweet cherry remain to be validated [74,75]. Despite unchanged ABA content at 124 dpi, such transcriptional adjustments may influence longer-term drought–pathogen interaction outcomes, given the known ABA–SA antagonism [15,16].

Physiologically, stress interactions were limited at the assessed time point, suggesting transcriptional buffering or effects emerging later. PCA supported cultivar-specific combined-stress programs, with cv. Bing driving separation along PC1, consistent with stronger reprogramming in more stress-susceptible genotypes [23,76]. Among contrasting DEGs, CAR1 homolog expression diverged between cultivars, consistent with differential immune regulation [77,78]. GIGANTEA (GI) was consistently overexpressed under combined stress in both cultivars; given its reported roles in circadian regulation, chloroplast function, and ABA-related processes, rather than a direct effector of downstream physiological processes [30,31,33,79]. GI represents a candidate link between drought and biotic signaling. However, in this study, the evidence remains correlational at the transcript level, and functional validation will be required to determine whether GI acts as a regulatory hub under combined stress. Finally, WRKY22, WRKY40, and WRKY53 were repressed under biotic stress alone but induced under combined stress in cv. Bing, supporting stress-specific transcriptional shifts likely driven by the added drought constraint [73,80,81,82].

## 4. Materials and Methods

### 4.1. Plant Material

The sweet cherry cvs. Bing and Santina differ in their susceptibility to *P. syringae* pv. *syringae*, with cv. Bing being more and cv. Santina being less susceptible based on field observations [6]. Both cultivars were grafted onto Gisela 12 (*P. cerasus* × *P. canescens*) and used in this study. One-year-old trees were purchased at a nursery in Chile (34°28′37″ S 70°58′44″ W). Sweet cherry cultivars were confirmed through a genetic analysis based on microsatellite markers [83].

The trees were kept in greenhouse for three months, at 25 °C to 27 °C, and 57–67% relative humidity, under drip irrigation (2 L h^−1^) at the Centro de Estudios Avanzados en Fruticultura CEAF (34°19′21″ S; 70°50′02″ W). On 6 December 2022, the plants were moved to a shaded acclimation area and subsequently transplanted to 20 L containers with a 1:1 peat/perlite mix supplemented with 6 g L^−1^ Basacote® Plus 9M (BASF, Ludwigshafen, Germany). The plants were transferred to the field on 6 January 2023. Basacote® Plus 9M is a polymer-coated, controlled-release fertilizer with a nominal nutrient release period of approximately 8–9 months, depending on temperature conditions. Its formulation consists of 16–8–12 (N–P_2_O_5_–K_2_O) with 2% MgO, supplied as a mixture of ammonium and nitrate nitrogen forms. In addition, Basacote^®^ Plus 9M contains micronutrients, including Fe (0.37%), Mn (0.06%), B (0.02%), Cu (0.05%), Zn (0.02%), and Mo (0.01%), provided in readily available forms. The fertilizer was applied according to the manufacturer’s recommendations for woody perennial crops.

### 4.2. Description of Treatments and Stress Application

To assess the effects of biotic, abiotic, and combined stresses, eight treatments were defined according to cultivar: two inoculation conditions (mock or *P. syringae* pv. *syringae*) with two irrigation regimes, well-watered (WW; plants maintained at field capacity) and water deficit (WD; irrigation reduced to induce moderate drought stress).

The treatments were defined as follows: under biotic stress conditions (WW with *P. syringae* pv. *syringae*), treatment T1 corresponded to cv. Santina trees and T3 to cv. Bing trees inoculated with *P. syringae* pv. *syringae* 11116B1, previously characterized as highly pathogenic. The bacterial strain was isolated by the University of Chile and confirmed by whole genome sequencing (GenBank accession number GCA_029383325.1). The mock under well-watered conditions were T2 (cv. Santina) and T4 (cv. Bing), both non-inoculated. Abiotic stress treatments (WD without *P. syringae* pv. *syringae*) included T6 for cv. Santina and T8 for cv. Bing. Finally, combined-stress conditions (WD with *P. syringae* pv. *syringae*) were represented by T5 (cv. Santina) and T7 (cv. Bing) (Table 1). Each treatment consisted of three biological replicates, with one individual tree per replicate, resulting in a total of 24 plants (8 treatments × 3 trees per treatment). Each tree represented an independent experimental unit throughout the study.

#### 4.2.1. Bacterial Isolate and Plant Inoculation

The bacterial inoculum was prepared from a pure stock stored at −80 °C in nutrient broth (NB; meat extract 0.3%, peptone 0.5%) with 15% glycerol. An aliquot of the culture was plated onto *Pseudomonas* agar F (PAF) supplemented with cycloheximide at 100 µg mL^−1^ and incubated at 26 °C for 16 h. A single colony was transferred to Luria–Bertani (LB) liquid medium [84] and incubated at 26 °C with shaking (100 rpm) for 12 h to promote an exponential phase growth. On the day before the inoculation, an aliquot of the bacterial culture was transferred to fresh LB medium and incubated at 26 °C with shaking until it reached an optical density (OD) of 0.1 at 600 nm, corresponding to approximately 10^8^ CFU mL^−1^.

The inoculation was carried out in greenhouse. For each tree, the shoot was wounded at a 45° angle between the third and sixth internodes from the apex using sterile razor blades, cutting to approximately half the diameter of the shoot. Immediately, 20 µL of either bacterial suspension or sterile distilled water (mock) was added to the wound, followed by two drops of glycerol in the surrounding area. The wound was then covered with parafilm to maintain moisture, support the stem, and promote healing [25].

#### 4.2.2. Irrigation Regimes

Irrigation was standardized prior to treatment application (WD). All plants were irrigated three times per week for approximately 45 min using two drippers per plant with a flow rate of 2 Lh^−1^ until saturation, and their weight was recorded when the water drainage was stopped [7,85]. This uniform irrigation regime was maintained until February 15, 2023 corresponding to 84 dpi with *P. syringae* pv. *syringae* 11116B1. Thereafter, differential irrigation was applied.

Each pot was watered to saturation, and after drainage (a proxy for field capacity), the pot was weighed to estimate field capacity (Wfc). Subsequently, the plants were irrigated three times per week. Before each irrigation, the pot was weighed using a digital scale. Transpired water (Ti) was calculated as the difference between the post-irrigation weight and the pre-irrigation weight from the previous watering. WW plants were watered to saturation, while WD plants received a reduced volume of water to maintain the fraction of transpirable soil water (FTSW) at the level of the fourth-lowest Ti.

Water deficit conditions were validated by measuring stem water potential (Ψ) and stomatal conductance (gs), targeting Ψ < −1 MPa and g_s_ ≤ 100 mmol m^−2^ s^−1^ [63,86]. Stem Ψ was measured using a pressure chamber (Model 1505D, PMS Instrument Company, Albany, OR, USA) on fully expanded, undamaged leaves located between nodes 7 and 15 from apex to base and fully exposed to sunlight. Prior to measurement, leaves were wrapped in damp paper towels, enclosed in aluminized plastic bags for at least 1 h while still attached to the plant, and then excised with a sterile razor blade. Leaves were transported in a cooler box and pressurized within five minutes of detachment. The petiole was positioned through the chamber lid, and nitrogen gas was used to pressurize the chamber. The pressure at which xylem sap appeared at the cut surface—visualized using a stereo microscope (Model XTL-101Bled, L&T Optics, Fuqing, China)—was recorded as Ψ. Midday Ψ (Ψmds) was measured between 11:15 and 13:45 h [7]. All experimental units (trees) were arranged in a completely randomized design. Before treatment application, pots were randomly positioned in the greenhouse and later in the field.

### 4.3. Infection Monitoring

Visual inspections were conducted throughout the experiment. To confirm bacterial infection, tissue samples were collected at 49 dpi from three positions relative to the inoculation site: P1 (leaf above the inoculation point), P2 (shoot zone above the inoculation point), and P3 (shoot zone below the inoculation point). Samples were surface-sterilized, macerated, and plated on PAF. Colonies were incubated for 48 h at 26 °C, counted, and confirmed by PCR using syrB and syrD primers [87]. At 124 dpi, RNA-seq reads were de novo assembled using CLC Genomics Workbench v23.1 and mapped to the *P. syringae* pv. *syringae* 11116B1 reference genome to confirm bacterial presence at the transcriptomic level.

### 4.4. Physiological and Biomass Measurements

#### 4.4.1. Leaf Gas Exchange

Leaf gas exchange measurements at 124 dpi were conducted between 11:15 h and 13:45 h, using a portable gas analyzer (CIRAS-3, PP Systems, Amesbury, MA, USA). Fully expanded, sun-exposed mature leaves, with incident PAR > 1400 µmol photons m^−2^ s^−1^, located near those used for Ψmds measurements, were selected. To determine net photosynthesis rate (A) and stomatal conductance (g_s_), leaves were acclimated in the cuvette for 60 to 90 s until steady state was reached. The CO_2_ concentration was adjusted to 400 µmol mol^−1^, and the light, humidity, and temperature were set to ambient conditions [7].

#### 4.4.2. Biomass

At 124 dpi, three biological replicates per treatment were harvested and oven-dried at 70 °C for 72 h to determine total aerial, root, leaf, and stem biomass (g DW per tree).

#### 4.4.3. Hormone Determination

Dry leaf tissue (80 mg) was collected at 1 and 124 dpi for phytohormone analysis. The quantification focused on ABA, jasmonic acid (JA), salicylic acid (SA), auxins (indole-3-acetic acid, IAA), and gibberellic acid (gibberellin A3, GA3). Samples were spiked with 50 ng of deuterium-labeled internal standards (2H6-ABA, 2H6-JA, 2H4-SA, 2H5-IAA, and 2H2-GA3) (OlChemIm, Olomouc, Czech Republic). The analysis was conducted at the National University of Río Cuarto (UNRC), Córdoba, Argentina. For liquid chromatography (LC) (Alliance 2695 LC Separation Module, Waters Corporation, New York, NY, USA), 10 µL of each sample was injected into a Waters Alliance 2695 LC system (Waters, USA) equipped with an autosampler and a Restek C18 column (2.1 × 100 mm, 5 µm; Bellefonte, PA, USA) maintained at 28 °C. The binary solvent system consisted of 0.2% acetic acid in LC-MS-grade water (solvent B) and 100% methanol (solvent A), with a constant flow rate of 200 µL min^−1^. A linear gradient elution profile was applied as follows: [t (min), % A]: (0, 40), (25, 80), followed by a 7 min re-equilibration period.

Mass spectrometry (MS) analysis was performed on a Micromass Quatro Ultima™ PT triple quadrupole mass spectrometer (Micromass, Manchester, UK) using a turbo electrospray ionization (ESI) source in negative ion mode. Instrumental parameters were set as follows: capillary voltage −3250 V, cone energy 35 V, RF1 lens 20, RF2 lens 0.3, source temperature 100 °C, desolvation temperature 350 °C, cone gas flow 100 L h^−1^, and desolvation gas flow 701 L h^−1^. The collision cell potential was set to 15 V, and the multiplier to 650. MS/MS parameters were optimized using standard solutions of each hormone. Quantification was performed using multiple reaction monitoring (MRM) to distinguish compounds with identical nominal masses. Data were analyzed using MassLynx™ 4.1 and QuanLynx™ 4.1 software (Micromass, Manchester, UK). Hormone levels were calculated based on calibration curves constructed with known concentrations of each hormone and their corresponding deuterated internal standards, obtained from Sigma (St. Louis, MO, USA).

### 4.5. Transcriptomic Analysis

#### 4.5.1. RNA Extraction and Library Construction

Tissue samples were collected from three independent biological replicates per treatment, sampling the area near the inoculation site. RNA-seq sampling was conducted at 124 dpi to target late-season, host-driven transcriptional adjustments under water deficit and following bacterial infection. For each biological replicate, approximately 100 mg of fresh stem tissue was collected from the area adjacent to the inoculation site. Each replicate sample originated from a different individual tree subjected to the same treatment, ensuring biological independence among replicates. Tissue samples were immediately frozen in liquid nitrogen and stored at −80 °C prior to RNA extraction.

Tissues were macerated using 1 mL of lysis solution and immediately frozen in liquid nitrogen. Total RNA was extracted using Spectrum™ Plant Total RNA Kit (Sigma-Aldrich, St. Louis, MO, USA) as per the manufacturer’s instructions. RNA integrity was evaluated by 1% agarose gel electrophoresis. RNA concentration and purity (R260/280) were measured with an Infinite^®^ 200 PRO NanoQuant spectrophotometer (Tecan Group, Männedorf, Switzerland). All column-purified RNA samples in this study showed acceptable purity (R260/280 ratios > 1.7). RNA-Seq paired-end libraries were constructed using Illumina TruSeq Stranded Total RNA with Ribo-Zero Plant Kit (Illumina, San Diego, CA, USA), generating paired-end reads of 151 nts.

#### 4.5.2. Reads Analysis and Mapping to Reference Transcriptome

Raw sequencing reads were processed using CLC Genomics Workbench version 23.1 (QIAGEN, Hong Kong, China). Quality control included trimming reads with an error probability > 0.01, discarding ambiguous reads containing more than two undefined nucleotides, and removing reads shorter than 50 nucleotides. Clean reads were mapped to *P. avium* cv. Satonishiki reference transcriptome (GenBank accession number GCA_002207925.1) [88], using the following parameters: similarity fraction ≥ 0.95, length fraction ≥ 0.8, insertion/deletion cost = 3, mismatch cost = 2, and unspecific match limit = 10. The reference transcriptome comprised 35,009 transcripts corresponding to 25,841 predicted protein-coding genes, and was selected due to its broad acceptance and high mapping efficiency reported in previous transcriptomic studies across diverse *P. avium* cultivars [25,45,89].

#### 4.5.3. Differential Gene Expression Analysis

Differential gene expression (DGE) analysis was performed using the Differential Expression for Two Groups tool in CLC Genomics Workbench v23.1, applying a multi-factorial negative binomial generalized linear model (GLM). Trimmed mean of M-values (TMM) normalization was used to correct for differences in library size among samples. Pairwise comparisons were performed between each treatment and the corresponding mock control of the same cultivar (T2 and T4): T1, T5, and T6 were compared to T2 (cv. Santina); T3, T7, and T8 were compared to T4 (cv. Bing). These comparisons allowed for the identification of differentially expressed genes in response to biotic, abiotic, and combined stresses within each cultivar. Additional comparisons were conducted between cultivars under equivalent treatment conditions to evaluate genotype-specific transcriptional responses. Significantly differentially expressed genes (DEGs) were identified using Benjamini–Hochberg false discovery rate (FDR) correction of 0.05 [90]. Transcripts with an absolute fold change ≥ 2.0 and an FDR-adjusted *p*-value ≤ 0.05 were considered significantly differentially expressed. To assess the quality and consistency of the data, housekeeping gene expression [38] was evaluated.

#### 4.5.4. Gene Ontology (GO) Annotation and Enrichment

Significantly expressed genes were functionally annotated using the Database for Annotation, Visualization and Integrated Discovery (DAVID) (https://david.ncifcrf.gov/home.jsp, accessed on 16 January 2025), using an EASE score threshold (Enrichment Analysis of Superset Entities) ≤ 0.05 [91]. Enrichment bubble plots were generated using the SRplot (http://www.bioinformatics.com.cn/srplot, accessed on 16 January 2025) [92]. Additional functional categories were retrieved using QuickGO (www.ebi.ac.uk/QuickGO, accessed on 17 January 2025) and UniProt (www.uniprot.org, accessed on 17 January 2025) to explore molecular functions, biological processes, and cellular components associated with DEGs.

#### 4.5.5. Validation of Differentially Expressed Genes by RT-qPCR

To validate RNA-seq results, eight DEGs showing significant expression differences between the mock and treatments were selected (Table S3). Primers were designed using Primer3 v4.1.0 (https://primer3.ut.ee/, accessed on 5 December 2024). Retro transcribed quantitative PCR (RT-qPCR) was conducted using Brilliant II SYBR Green QPCR Master Mix (Agilent Technologies, Santa Clara, CA, USA) on a StepOne Real-Time PCR System (Applied Biosystems, Waltham, MA, USA), following the manufacturer’s instructions. Each 15 µL reaction contained 12 ng of cDNA and 500 nM of each primer, and reactions were performed in technical triplicate. Amplification conditions consisted of an initial denaturation at 95 °C for 15 min, followed by 40 cycles of 95 °C for 15 s and 60 °C for 1 min. The RNA polymerase II subunit gene (RPII) was used as an internal reference for normalization (Table S3). Relative expression levels were calculated using the 2^−ΔΔCt^ method with primer efficiency correction [93]. The correlation between qPCR and RNA-seq expression values was assessed using Spearman’s rank correlation test, with results expressed as correlation coefficients (r) and associated *p*-values.

### 4.6. Statistical Analysis

A 2 × 2 × 2 factorial design was implemented to evaluate the effects of cultivar (Santina and Bing), irrigation regime (well-watered, WW; water deficit, WD), and inoculation treatment (*P. syringae* pv. *syringae* or sterile water) on sweet cherry trees. This experimental setup resulted in eight treatment combinations, each consisting of three biological replicates (one tree per replicate), arranged in a completely randomized design.

Prior to analysis, data were evaluated for normality using the Shapiro–Wilk test and for homogeneity of variances using Levene’s test. Physiological variables, including stomatal conductance (g_s_), net photosynthetic rate (A), and biomass, were analyzed using generalized least squares (GLS) models. The fixed effects included cultivar, irrigation regime, inoculation treatment, and interactions. When significant main effects or interactions were detected, adjusted means were compared using Fisher’s least significant difference (LSD) test at α = 0.05. Water-use efficiency (WUE) was analyzed using a non-parametric Kruskal–Wallis test, and results are reported using the corresponding H statistic.

Colony counts at 49 dpi were analyzed separately for each cultivar (Bing and Santina) and sampling zone. For each combination of cultivar and sampling zone, colony counts were modeled using generalized linear models with a negative binomial error distribution and a log link function.

Hormone concentration data were analyzed separately for each sampling time point (1 and 124 dpi). Gibberellic acid (GA_3_) met the assumptions of normality and homoscedasticity and was therefore analyzed using linear models within the factorial framework. When significant effects were detected, treatment means were compared using Fisher’s least significant difference (LSD) test at the 5% significance level.

Salicylic acid (SA) data did not meet normality assumptions in the original scale but satisfied model assumptions after log_10_ transformation. Log-transformed SA values were analyzed using linear models, and Fisher’s LSD test (*p* < 0.05) was applied for post hoc comparisons when significant main effects were detected.

Indole-3-acetic acid (IAA), jasmonic acid (JA), and abscisic acid (ABA) data did not meet assumptions. These hormones were therefore analyzed using generalized linear models (GLM) with a Gamma error distribution and log-link function. For these datasets, model significance was assessed based on the fitted GLM results.

The images were processed using high-end scaler software [94].

## 5. Conclusions

At 124 dpi, combined *P. syringae* pv. *syringae* infection and water deficit elicited non-additive, cultivar-specific transcriptional responses in sweet cherry. Cv. Bing exhibited a more extensive and novel transcriptional reprogramming, whereas cv. Santina showed greater integration of abiotic stress-related signaling. These contrasting strategies were reflected in divergent molecular adjustments: cv. Bing strongly repressed photosynthesis-related processes while activating defense- and hormone-associated pathways, whereas cv. Santina displayed limited photosynthetic repression together with selective modulation of lignin and diterpenoid biosynthesis, consistent with differential regulation of structural and chemical defenses.

Taken together, these findings provide a molecular rationale for practical applications in cherry breeding and management under climate change. For breeding programs, the recurrent induction of GIGANTEA as a candidate integrator of biotic and abiotic stress signaling, together with the contrasting regulation of WRKY40, suggests that selecting genotypes with efficient stress-signal integration, capable of maintaining protein homeostasis and photosynthesis (as observed in cv. Santina), may be more effective than selecting for single-stress resistance traits. Furthermore, the broad downregulation of cuticular wax and suberin biosynthesis genes in the susceptible cv. Bing under combined stress highlights a critical vulnerability warranting further investigation. Consequently, orchard management strategies in warming climates should prioritize precision irrigation not only to sustain plant water status, but also to mitigate stress-driven transcriptional repression of structural defenses, thereby enhancing long-term resilience to combined biotic and abiotic stress under climate change scenarios.

## Figures and Tables

**Table 1:** Experimental design and treatment definitions used to evaluate biotic, abiotic, and combined stress responses in sweet cherry.

Treatment	Cultivar	Inoculation Condition	Irrigation Regime	Stress Category
T1	Santina	*Pseudomonas syringae* pv. *syringae* (Pss11116B1)	Well-watered (WW)	Biotic stress
T2	Santina	Mock (sterile water)	Well-watered (WW)	Control
T3	Bing	*Pseudomonas syringae* pv. *syringae* (Pss11116B1)	Well-watered (WW)	Biotic stress
T4	Bing	Mock (sterile water)	Well-watered (WW)	Control
T5	Santina	*Pseudomonas syringae* pv. *syringae* (Pss11116B1)	Water deficit (WD)	Combined stress
T6	Santina	Mock (sterile water)	Water deficit (WD)	Abiotic stress
T7	Bing	*Pseudomonas syringae* pv. *syringae* (Pss11116B1)	Water deficit (WD)	Combined stress
T8	Bing	Mock (sterile water)	Water deficit (WD)	Abiotic stress

## Data Availability

Data are available from the authors upon request.

## Supplementary Materials

The following supporting information can be downloaded at: https://www.mdpi.com/article/10.3390/plants15030450/s1, Table S1: Summary of RNA-seq library sequencing metrics; Table S2: Differential expression of salicylic acid-related pathway genes; Table S3: List of DEGs selected for qPCR validation.

## Abbreviations

The following abbreviations are used in this manuscript:

ABA	Abscisic acid
ACT	Actin (reference gene)
A	Net CO_2_ assimilation rate
bp	Base pairs
bZIP	Basic leucine zipper transcription factor
CC	Cellular Components (GO category)
CFU	Colony-forming units
CRD	Completely randomized design
cv.	Cultivar
DEGs	Differentially expressed genes
dpi	Days post-inoculation
DW	Dry weight
E	Transpiration rate
ESI	Electrospray ionization
ETI	Effector-triggered immunity
FDR	False discovery rate
FTSW	Fraction of transpirable soil water
GA_3_	Gibberellic acid (gibberellin A3)
GC	GC content (guanine–cytosine percentage in sequencing)
GI	GIGANTEA gene
GLM	Generalized linear model
GO	Gene Ontology
gₛ	Stomatal conductance
H	Kruskal–Wallis test statistic
HDA	Histone deacetylase
IAA	Indole-3-acetic acid
JA	Jasmonic acid
KEGG	Kyoto Encyclopedia of Genes and Genomes
LB	Luria–Bertani medium
LC	Liquid chromatography
LMM	Linear mixed-effects model
LSD	Least significant difference
MRM	Multiple reaction monitoring
MS	Mass spectrometry
Myb	Myb-related proteins/Myb-like transcription factors
OD	Optical density
PAF	Pseudomonas agar F
PAL	Phenylalanine ammonia-lyase
PAR	Photosynthetically active radiation
PCA	Principal component analysis
PCR	Polymerase chain reaction
Pss	*Pseudomonas syringae* pv. *syringae*
PTI	PAMP-triggered immunity
Q20, Q30	Sequencing quality score metrics
RNA-seq	RNA sequencing
RPII	RNA polymerase II (reference gene)
ROS	Reactive oxygen species
rpm	Revolutions per minute
SA	Salicylic acid
SD	Standard deviation
T1–T8	Stress treatments, as defined in the methods
TMM	Trimmed mean of M-values normalization
Ti	Transpired water
TF	Transcription factor
Ψ	Stem water potential
Ψmds	Midday stem water potential
WD	Water deficit
WW	Well-watered
WUE	Water-use efficiency
