# Supplementary material for: Contrasting Response of Santina and Bing Sweet Cherry Cultivars Under Combined Biotic and Abiotic Stress
Source: Plants (Basel). 2026 Feb 1;15(3):450. doi: 10.3390/plants15030450 (PMC12899965; doi:10.3390/plants15030450)
Supplement: Supplementary file 1 [file plants-15-00450-s001.zip › plants-4076718-supplementary.pdf]

Supplementary Table 3. List of DEGs selected for qPCR validation.

Gene ID	Protein names	Left
XP_021811685	Chlorophyll a-b binding protein, chloroplastic	CATTCAAGATGGGCCATGCT
XP_021811722	Xyloglucan endotransglucosylase/hydrolase (EC 2.4.1.207)	GGTCTCAATGGTGCTTGTGG
XP_021801566	Pathogenesis-related protein PR-4	CGCAGCAAATATGGATGGACT
XP_021801743	Sucrose synthase (EC 2.4.1.13)	AGAGGCTGAGGAGTTTGGTC
XP_021805326	DNA-directed RNA polymerases II, IV and V subunit 3-like	CTCATCCCATTGACCTCCGA
XP_021810623	glucan endo-1,3-beta-D-glucosidase	AGCTTCAAGGCCTCACTGAT
XP_021833124	(-)-alpha-pinene synthase-like	AAGAAGCACTGGAACGCATG
XP_021803569	anthocyanidin synthase	CAGACCCAAGGAAGAGCTCA
XP_021821998	Protein trichome birefringence-like 41	CCAGCTGCCCTTTCATTGAG

Right	Product length	
TAGTCTAGCCCACCCTCACT	134	Foto
AGTGTGAGAAGCTGTCCTCC	135	Wall
CTCCATTGCTGCACTGATCC	142	Defb
TCCATCTGAACTGACCCGTC	143	Sucr
GTTTGGTCGGAATGGCACTT	129	RPII
GGATGGCTGGGAGGAGATAC	152	Glu
CTTCTCCATGCACCCTCAGA	236	PIN
CACTGCTGCCTTGTTCAACT	157	Ant
TGAGGCTTAGAGAGTCCCCT	180	Tri
